# A Genomic Snapshot of Antibiotic-Resistant*Enterococcus faecalis* within Public Hospital Environments in South Africa

**DOI:** 10.1155/2023/6639983

**Published:** 2023-06-12

**Authors:** Christiana O. Shobo, Daniel G. Amoako, Mushal Allam, Arshad Ismail, Sabiha Y. Essack, Linda A. Bester

**Affiliations:** ^1^Antimicrobial Research Unit, College of Health Sciences, University of KwaZulu-Natal, Durban 4000, South Africa; ^2^School of Laboratory Medicine and Medical Science, Department of Medical Microbiology, University of KwaZulu-Natal, Durban 4000, South Africa; ^3^Biomedical Resource Unit, School of Laboratory Medicine and Medical Sciences, College of Health Sciences, University of KwaZulu-Natal, Durban 4000, South Africa; ^4^Department of Genetics and Genomics, College of Medicine and Health Sciences, United Arab Emirates University, Al Ain 15551, UAE; ^5^Sequencing Core Facility, National Institute for Communicable Diseases, National Health Laboratory Service, Johannesburg 2131, South Africa; ^6^Department of Biochemistry and Microbiology, Faculty of Science, Engineering and Agriculture, University of Venda, Thohoyandou 0950, South Africa

## Abstract

Enterococci are among the most common opportunistic hospital pathogens. This study used whole-genome sequencing (WGS) and bioinformatics to determine the antibiotic resistome, mobile genetic elements, clone and phylogenetic relationship of *Enterococcus faecalis* isolated from hospital environments in South Africa. This study was carried out from September to November 2017. Isolates were recovered from 11 frequently touched sites by patients and healthcare workers in different wards at 4 levels of healthcare (A, B, C, and D) in Durban, South Africa. Out of the 245 identified *E. faecalis* isolates, 38 isolates underwent whole-genome sequencing (WGS) on the Illumina MiSeq platform, following microbial identification and antibiotic susceptibility tests. The *tet(M)* (31/38, 82%) and *erm(C)* (16/38, 42%) genes were the most common antibiotic-resistant genes found in isolates originating from different hospital environments which corroborated with their antibiotic resistance phenotypes. The isolates harboured mobile genetic elements consisting of plasmids (*n* = 11) and prophages (*n* = 14) that were mostly clone-specific. Of note, a large number of insertion sequence (IS) families were found on the IS3 (55%), IS5 (42%), IS1595 (40%), and Tn3 transposons the most predominant. Microbial typing using WGS data revealed 15 clones with 6 major sequence types (ST) belonging to ST16 (*n* = 7), ST40 (*n* = 6), ST21 (*n* = 5), ST126 (*n* = 3), ST23 (*n* = 3), and ST386 (*n* = 3). Phylogenomic analysis showed that the major clones were mostly conserved within specific hospital environments. However, further metadata insights revealed the complex intraclonal spread of these *E. faecalis* major clones between the sampling sites within each specific hospital setting. The results of these genomic analyses will offer insights into antibiotic-resistant*E. faecalis* in hospital environments relevant to the design of optimal infection prevention strategies in hospital settings.

## 1. Introduction

The surveillance of hospital environments can be a useful tool to better understand the opportunistic microbial communities within the hospital (Comar et al., 2019), to identify the source of an outbreak [1], and to evaluate the efficacy of environmental disinfection or other infection prevention and control measures [2]. Inadequate control practices have played a significant role in the dissemination, persistence, and intra and interhospital spread of drug-resistant organisms. Regrettably, good clinical trials comparing different approaches to and the impact of infection prevention and control interventions on the control of drug-resistant bacteria in hospitals and other healthcare facilities are minimal [3, 4]. Accurate identification of resistant bacterial reservoirs and modes of transmission helps to inform such interventions.

The latest successes in tracing worldwide epidemics [5] and nosocomial outbreaks [6] have been attributed to whole-genome sequencing (WGS). Genomic comparison has aided our understanding of the evolution and spread of infectious agents. Comparative genomic analyses have been made possible through the use of WGS, showing the extent of genomic variation, which may result in varied phenotypes, thus expanding our understanding of diverse genomic determinants such as antibiotic resistance genes and their mobile genetic elements in bacterial species [6, 7].

*Enterococcus faecalis (E. faecalis)* is a good indicator bacterium in hospital environment monitoring, and being Gram-positive cocci, these opportunistic pathogens not only form noxious biofilms on implanted medical devices and catheters but also they cause abdominal infections, urinary tract infections, surgical site wound infections, bacteremia, endocarditis, and burn wound infection [8]. Antibiotic resistance is either intrinsic or through sporadic mutation or through the acquisition of foreign genetic material, by horizontal gene exchange occurring with the aid of mobile genetic elements plasmids, prophages, and insertion sequences [9, 10]. Difficulties in treating *E. faecalis* and *E. faecium* (the most prevalent species in human) have emerged due to acquired resistance, predominantly multidrug resistance to universally used drugs as well as vancomycin [11]. A number of previous surveillance studies involving *E. faecalis* in Africa have focused either on wastewater treatment plants (WWTPs) and hospital effluent but not on the internal hospital environment [12, 13]. Moreover, in South Africa, studies on the contamination of *E. faecalis*, using high discrimination resolution typing, are scarce. This study, therefore, uses WGS in delineating the resistome, mobile genetic elements, clones and phylogenomic relationship of *E. faecalis* isolated from the hospital environment in places frequently touched by patients and healthcare workers at four different levels of healthcare in the metropolitan city of Durban, South Africa.

## 2. Materials and Methods

### 2.1. Ethical Approval

Ethical clearance was received from the Biomedical Research Ethics Committee of the University of KwaZulu-Natal (Ref. BE606/16). The study was also registered with the Health Research and Knowledge Management database (HRKM 098/17) of the KwaZulu-Natal Provincial Health Research Ethics Committee.

### 2.2. Enterococcus Collection

The isolates described here at a genomic level were part of a previously reported study and appeared in [14]. Briefly, four public hospitals situated in the eThekwini region in Durban, South Africa were sampled and referred to as A, B, C, and D, representing central, tertiary, regional, and district facilities, respectively. Each hospital is classified according to the Department of Health and depends on the level of service it can provide to the community. The sample sites viz. ward telephones, ventilators, blood pressure apparatus, patient files, drip stands, sinks, occupied beds, unoccupied beds, nurses' tables, mops, and the door handle of the linen room were situated in the general ward, intensive care unit, and paediatrics. Suspected colonies were phenotypically identified using API 20 Strep kits (bioMerieux SA, Marcy I ‘Etoile, France). *Staphylococcus aureus* ATCC 29213 and *E. faecalis* ATCC 29212 served as controls. Molecular confirmation of isolates was also previously described [15]. To summarise, of the 620 samples taken, 295 *Enterococcus* spp. were obtained, of which 245 were confirmed as *Enterococcus faecalis* via phenotypic and molecular assays. A subsample of 38 *E. faecalis* isolates was selected for genotypic characterization by WGS and bioinformatics analysis (Table 1).

### 2.3. Antibiotic Susceptibility Testing (AST)

The antibiotic susceptibility of the isolates was determined using the Kirby-Bauer disk diffusion method (Hudzicki and Kirby-Bauer disk diffusion susceptibility test protocol) [16]. According to Clinical and Laboratory Standards Institute (CLSI) guidelines [17], the following antibiotics were used: ampicillin (10 *μ*g), penicillin (5 *μ*g), vancomycin (30 *μ*g), teicoplanin (30 *μ*g), erythromycin (15 *μ*g), tetracycline (30 *μ*g), ciprofloxacin (5 *μ*g), levofloxacin (5 *μ*g), nitrofurantoin (300 *μ*g), chloramphenicol (30 *μ*g), linezolid (30 *μ*g), and rifampicin (5 *μ*g). All discs were sourced from Oxoid (Basingstoke, United Kingdom). *Staphylococcus aureus* ATCC 25923 was used as the control. High-level aminoglycoside resistance was determined using gentamicin (120 *μ*g) and streptomycin (300 *μ*g) discs on Mueller-Hinton agar (Oxoid, Hampshire, England) with *E. faecalis* ATCC 29212 as the control isolate.

### 2.4. DNA Isolation, Genome Sequencing, Assembly, and Annotation

Genomic DNA (gDNA) was extracted using GenElute bacterial genomic DNA kit (Sigma–Aldrich, St. Louis, Missouri, United States) according to the manufacturer's instructions. The quantification of extracted gDNA was determined on a Nanodrop ND1000 spectrophotometer (Thermo Scientific, Waltham, USA) and Qubit 2.0 fluorometer (Invitrogen, Oregon, USA) and verified on an agarose gel electrophoresis. Multiplexed paired-end libraries (2 × 300 bp) were prepared using the Nextera DNA Flex sample preparation kit (Illumina, San Diego, California, United States) and sequences determined on an Illumina MiSeq platform with 100x coverage at the National Institute of Communicable Diseases Sequencing Core Facility, South Africa. The resulting raw reads were checked for quality, trimmed, and de novo assembled into contigs using the CLC Genomics Workbench version 10.1 (CLC, Bio-QIAGEN, Aarhus, Denmark). Default parameters were used for all software unless otherwise specified. The CheckM tool version 0.9.7 [18] was used to verify that the sequence reads were not from mixed species using lineage-specific marker sets from other genetically well-characterised closely related *E. faecalis* isolates. The *de-novo*-assembled reads were uploaded in GenBank and annotated using National Centre for Biotechnology Information (NCBI) prokaryotic genome annotation pipeline and Rapid Annotations using Subsystems Technology (RAST) 2.0 server [19].

### 2.5. WGS-Based Molecular Typing of *E. faecalis* Isolates

Multilocus sequence typing (MLST) was performed in silico using the WGS data online platform tool MLST 1.8 [20] which also predicted the allelic profiles of the seven housekeeping genes, *aroE, gdh, gki, gyd, psts, xpt, and yqil* of *E. faecalis* as described previously [21].

### 2.6. Phylogenomic Analysis of *Enterococcus faecalis* Isolates

The de-novo-assembled contigs were uploaded, and the analysis was submitted to CSI (called SNPs & Infer) Phylogeny-1.4 (https://cge.cbs.dtu.dk/services/CSIPhylogeny-1.2), an online service which identifies single-nucleotide polymorphism (SNPs) from WGS data, filters and validates the SNP positions, and then infers phylogeny based on concatenated SNP profiles [22]. The pipeline was run with default parameters: a minimal depth at SNP positions of 10 reads, a minimal relative depth at SNP positions of 10%, a minimal distance between SNPs of 10 bp, a minimal *Z-score* of 1.96, a minimal SNP quality of 30, and a minimal read mapping quality of 25. A bootstrapped with 100 replicate indicators was applied to identify recombined regions and provide phylogenetic accuracy in groups with little homoplasy. The figtree (https://tree.bio.ed.ac.uk/software/figtree/) was used to edit and visualise the phylogenetic tree. The phylogeny was visualised alongside metadata for isolate demographics (including hospital, source, ward), sequence type, and antibiotic resistome using Phandango [23] to provide a comprehensive analysis to understand the complex intraclonal spread within each specific hospital setting using the generated phylogenomic tree.

### 2.7. Genomic Identification of the Antibiotic Resistome and Mobile Genetic Elements (MGEs)

In the bacterial analysis pipeline, ResFinder [24] was used to annotate and identify antibiotic-resistant genes using default parameters (threshold ID of 90% and a minimum length of 60%). Plasmid replicons were predicted through PlasmidFinder [25] (https://cge.cbs.dtu.dk/services/PlasmidFinder/). The PHAge Search Tool (PHAST; https://phast.wishartlab.com/) [26] server was used for the identification, annotation, and visualization of prophage sequences. The assembled genomes were further analysed for insertion sequences and transposons using ISFinder (https://isfinder.biotoul.fr/) [27]. RAST SEEDVIEWER (https://rast.nmpdr.org/seedviewer.cgi) [28] and Integrall database (https://integrall.bio.ua.pt/) [29] were also used to annotate and identify the investigated genomes for integrons and associated gene cassettes.

### 2.8. Data Availability

The raw read sequences and the assembled whole-genome contigs have been deposited in GenBank. The data are available under project number **PRJNA523601**.

## 3. Results

### 3.1. Prevalence of *Enterococcus feacalis* on Frequently Touched Surfaces in the Hospital Environment

As shown by [11], a total of 83.1% (245/295) *E. faecalis* were collected, distributed as 11.0% (27/245) from the district hospital with high rate isolated from the nurse's table (7) and door handles (8), 34.7% (85/245) from the regional hospital, occupied beds (13), mop (14), and nurse's table (12) have a high contamination rate. In the tertiary hospital, 34.7% (85/245), the phones (16), mops (11), and occupied beds (9), has the highest contamination isolation rate.

From the central hospital, 19.6% (48/245) samples were isolated with the sites with the highest contamination rates being the occupied beds (12) and mops (8) with 30.2% each. However, this outcome differed between hospital levels; e.g., in district hospitals, the door handles (27.6%), nurses' tables (24.1%), and mops (20.6%) were the most contaminated. In comparison, the regional hospital's most contaminated sites were mops and occupied beds (13.8% each) and again also the nurse's tables (11.9%).

For the tertiary hospital, it was the ward phones (14.4%) and mops (10.8%). The central hospital showed a similar outcome as the regional hospital with the highest contaminated sites as occupied beds (22.2%), nurses' tables, and mop (20.3% each). Looking at the highest contaminated sites, the risk of mops as vehicles of contamination and probable cross-contamination within the hospital environment is highlighted.

### 3.2. Vancomycin Susceptibility

The authors of [11] described the antibiotic susceptibility testing, and these revealed that none of the 245 identified *E. faecalis* isolates was vancomycin-resistant (VRE). However, a total of thirty-eight (38) were of intermediate susceptibility to vancomycin and were selected for genotypic characterization by WGS and bioinformatics analysis (Table 1). These 38 vancomycin-intermediate isolates were predominantly resistant to tetracycline (*n* = 31, 81%) followed by resistance to erythromycin (*n* = 18, 47%). A small number of isolates showed aminoglycoside resistance (gentamicin (*n* = 4) and streptomycin (*n* = 6)). The majority of the isolates were susceptible to ampicillin, penicillin, teicoplanin, and levofloxacin while all isolates were susceptible to nitrofurantoin (Table S2)

### 3.3. WGS-Based Species Confirmation and Molecular Typing

The identification of *E. faecalis* isolates was confirmed with generated genomic data via the global platform for genomic surveillance (Pathogenwatch). MLST analyses (ST) revealed that the *E. faecalis* in the provincial public healthcare facilities were multiclonal belonging to 15 different STs with 6 major STs belonging to ST16 (*n* = 7), ST40 (*n* = 6), ST21 (*n* = 5), ST126 (*n* = 3), ST23 (*n* = 3), and ST386 (*n* = 3) (Table 1), with diverse allelic profiles. Moreover, one isolate (2SIL2) belonged to a newly defined ST bearing a novel allele (ST922) [30].

### 3.4. Antibiotic Resistance Profiles and Resistance Genes of *E. faecalis* Isolates

The phenotypic resistance profiles displayed by the isolates are shown in Table S1. In total, 14 antibiotic resistance genes (ARGs) and variants were detected (Table 1). There were no specific differences in the resistome with regard to their hospital levels and wards. The frequency of ARGs ranged between 2–13 genes, with fifteen isolates carrying 3 resistance genes. Acquired ARGs confer resistance to tetracycline (*tet(M)* and *tet(L)*), macrolide-lincosamide-streptogramin B (MLS_B_) (*erm(B)* and *mphD*), aminoglycosides (*sat4A, aph3-lll, ant6-la, aac6-aph2*), trimethoprim-sulfamethoxazole (*dfrG* and *dfrK*), and phenicols (*catA* and *optrA*) were found in the isolates as shown in Table 1. The *tet(M)* and *erm(B)* genes were found in 82% (31/38) and 42% (16/38) of the isolates, respectively. The *dfrG* gene predominately caused resistance to trimethoprim–sulfamethoxazole (Tables 1 and S2). In some cases, the phenotypes were not corroborated by ARGs, as some isolates expressed resistance to antibiotics in the absence of the associated resistance determinants (Tables 1 and S1).

### 3.5. WGS Detection of Mobile Genetic Elements

WGS analysis revealed 11 different plasmid replicons from seven *rep* families that occurred in different combinations in the *E. faecalis* isolates (Table 2). pTEF2 (rep9), pTEF3 (repUS13), pAD1 (rep9), and pEFC1 (rep6) were the most predominant replicon types occurring in 37% (14/38), 34% (13/38), 34% (13/38), and 24% (9/38) isolates, respectively. Of note, two isolates 2SIL2 and 2SPJ101 from hospital D concomitantly harboured unique plasmid replicons (pk214 (rep7), pEFR (rep11), pPD1 (rep9), pRE25 (rep2), pUB110 (repUS14), pKH7 (rep7)) that were absent in the other isolates (Table 2). Eight (21%) of the isolates did not possess any plasmid replicons. The replicons harboured by the isolates were clonally related. For instance, the major replicon pTEF2 (rep9) was harboured by isolates belonging to ST21, while the replicon set pTEF3 (repUS13), pAD1 (rep9), and pEFC1 (rep6) were harboured in ST40 isolates. Furthermore, most of the isolates (*n* = 5) belonging to ST16 lacked plasmids.

The prophage analysis revealed that all isolates hosted at least one intact bacteriophage except for three isolates belonging to different STs (Table 2). The predominant intact bacteriophages found were the Entero_phiFL1A (*n* = 16, 42%), Entero_phiFL3A (*n* = 6, 16%), Entero_vB_IME197 (*n* = 6, 16%), and Entero_phiEf11 (*n* = 5, 13%). Four prophages were identified in one *E. faecalis* ST16 (3UPF4) strain isolated from the mop of a paediatric ward in hospital B with a peculiar bacteriophage (Lactoc_PLgT_1). The isolates 1C1H3, 1MPD4, 2U1K2, and 2UPF3 from different hospitals hosted 3 prophages. The prophages harboured by the isolates were clonally related (Table 2).

A myriad of FAMILIES was found in the isolates with no association with respect to the hospital and ward. The 5 major FAMILIES were IS3 (predicted to be linked with *Enterococcus faecium/Streptococcus agalactiae* source), IS5 (predicted to be associated with *Cyanotheca sp.* source), IS1595 (predicted to be linked with *Bacillus subtilis*), ISL3 (predicted to be linked with *Streptococcus mutans/thermophilus*), and IS607 (predicted to be linked with both *Campylobacter sp.* and *Virus NY2A*) (Table S3). The transposase (Tn3) linked to *Bacillus thuringiensis* was found in 7 of the isolates identified from different sources (Table S3). All isolates lacked integrons and their associated gene cassettes.

### 3.6. Phylogenomic and Metadata Analysis

**Figure 1:**
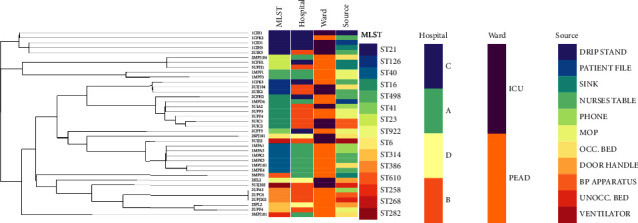
The whole-genome MLST phylogenomic branch and metadata of isolate information (including isolate identity, hospital, source, and ward) and WGS *in-silico typing* (sequence type and antibiotic resistome) coupled using Phandango (https://jameshadfield.github.io/phandango/) *E. faecalis* isolates at different level of care in Durban, South Africa. The linking lines in the phylogenetic tree differentiate between the different clades. Metadata annotations show that there were generally distinct major sequence types between the 4 hospital environments; however, within each hospital, there was the spread of these major clones between different sources in the wards.

A phylogenetic tree reconstructed to analyse the genetic relationships between the isolates revealed a high divergence of isolates according to the different levels of care (Figure 1). For instance, each hospital was generally associated with specific dominant clones (i.e., ST40 and ST498 were mostly found in hospital A; ST16, ST126, and ST386 were found in hospital B; and ST21 was predominately found in hospital C (Table 1 and Figure 1)).

The phylogenomic tree coupled with metadata visualization analysis provided a more in-depth insight into the characteristics and distinctions between isolates and revealed the intraclonal spread of *E. faecalis* strains between different sources within the same hospital (Figure 1). Specifically, ST21 was found on the drip stand, patient file, sink, and nurse table in both ICU and paediatric wards of hospital C. Similarly, ST40 was found on the phone, patient files, mops, occupied bed, and nurse tables of the paediatric ward hospital A. The ST16 clone was isolated from the mop (paediatric ward), phone, and BP apparatus (ICU) of hospital B. More so, ST386 was linked with the phone, BP apparatus, and unoccupied bed in the paediatric ward of hospital B, while ST126 was found with the occupied bed and nurses table in the ICU of the same hospital. The prophage Entero_vB_IME197 was mostly found among all ST21 strains and one ST41 strain (2CPF3) that derived from the regional hospitals; however, another ST21 strain (2UIK3) from a tertiary hospital also carried this prophage. The same prophage has been reported in an *E. feacium* strain belonging to ST 179 sourced from a wastewater plant (Mbanga et al., 2021). It has been reported that for *Enterococcus* species, it is more likely that isolates with common phage-related genes share common environments or a specific niche (Bonacina et al., 2016). Similarly, it could be seen with prophage Entero_phiFL1A which were predominantly sourced from the central hospital. Lacto 98201 and Lacto 63301 are Lactococcus protein-related phages, while Lactob _PLE2 is a Lactobacillus protein-related phage (Bonacina et al.,2016).

## 4. Discussion

In line with the global trend, reports on bacterial contamination in hospital environments are increasing in Africa across all sectors [31], and *E. faecalis* is one of the most common enterococcal species isolated from the hospital environment. This is evident from our results where *E. faecalis* (*n* = 245) was the most prevalent organism compared to *E. faecium* (*n* = 50). *E. faecalis* is recognised as an important hospital-associated pathogen responsible for approximately 80–90% of cases reported in hospital settings, followed by 5–10% *E. faecium* [32] and hence has been placed in the category of pathogens posing a major threat to healthcare systems [33]. Furthermore, *E. faecalis* represents a major infection prevention attributed to their ability to persist for long periods on hands and remain viable on environmental surfaces (inanimate surfaces) due to their microbial structure thus, which can serve as a reservoir for ongoing transmission in the absence of regular decontamination [34]. Additionally, *E. faecalis* possesses the ability to acquire additional resistance through the transfer of mobile genetic elements such as plasmids, prophages, and insertion sequences [35, 36]. The acquisition of resistance and genetic elements poses a therapeutic challenge.

The WGS results showed that none of the *E. faecalis* harboured vancomycin-resistant genes, which corroborated with the phenotypes [11]. This affirms the view of Ellington et al. on the role of WGS in antimicrobial susceptibility testing of bacteria for the explanation of phenotypic results for samples [37] and further confirms WGS as a more discriminatory tool to infer antibiotic susceptibility compared to relying fully on phenotypic testing alone. Additionally, the discordance between the phenotype and the genotype in *Enterococcus spp.* could be due to impaired penetration of the antibiotic, hetero-resistance, or limitations of the current traditional phenotypic (antimicrobial susceptibility testing) methods [38–40]. More so, while the presence of specific resistance genes suggests a potential mechanism for antibiotic resistance, it is important to note that other factors, such as gene expression, regulatory elements, and efflux pumps, can influence the observed phenotypic resistance [38, 40]. The majority of the isolates were susceptible to ampicillin, penicillin, teicoplanin, levofloxacin, and nitrofurantoin confirming their use as treatment options in South Africa, particularly ampicillin (the drug of choice for *E. faecalis* infections) [41].

In our result, tetracycline demonstrated reduced susceptibility to *E. faecalis* mediated mostly by the ribosomal protection protein, *tet(M)* [10, 42]. This was consistent with previous studies that found *tet(M)* as the dominant gene causing tetracycline resistance in *E. faecalis* isolates across all one-health sectors (human-animal-environment interface) [31]. For instance, in a 2014 hospital-based study in China by Jia et al. [43], tet*(M)* was found to cause tetracycline-resistant*E. faecal* isolates. Similarly, Said et al. [44] also detected tet*(M)* as 96.1% of all tetracycline-resistant*Enterococcus* isolates in Egypt. However, the tetracycline resistance exhibited by the 2SIL2 isolates was mediated by both the ribosomal-protection gene [*tet(M)*] and the active-efflux gene [*tet(L)*]. This indicates the significant role played by efflux pumps in mediating antibiotic resistance [45]. The low prevalence of the tet*(L)* was not unusual and pointed to the fact that ribosomal protection protein is the main mechanism of tetracycline-resistant*E. faecalis* isolates. Our result showed that a moderate level of erythromycin resistance was mediated by *erm(B)* genes which are the most common mechanism of resistance reported for the macrolide class of antibiotics in Africa [31] and globally [10, 46] for *Enterococcus*.

High levels of resistance to aminoglycosides have developed since its clinical utility for achieving bactericidal synergism in combination with cell wall-active agents, which is important in the treatment of enterococcal endocarditis [47]. This level of resistance is owing to the appearance of aminoglycoside-modifying enzymes and contradicts the synergistic advantage of the combinations in the clinical setting. The gene encoding the most known enzyme conferring resistance to aminoglycosides excluding streptomycin is *aac-6′-Ie-aph-2*″, typically discovered inside Tn4001 in staphylococci and other variants in enterococci [48]. From our result, only a small number of isolates showed aminoglycoside resistance across the different levels of care, which corresponded to the aminoglycoside-modifying enzymes found. However, these isolates exhibited high-level resistance encoding a set of enzymes (*sat4A, aph3-lll, ant6-la, aac6-aph2*) although this was not unusual as some *Enterococcus* spp. are known to produce low-level resistance to aminoglycosides by limiting drug uptake, which is associated with proteins involved in electron transport [49]. Furthermore, although the OptrA gene associated with linezolid resistance was detected in only one isolate (2SIL2); however, it was unexpressed as the isolate was susceptible to linezolid (Tables 1 and S2). This finding was similiar to a study conducted in China by Li et al. (2020), where the OptrA gene was identified in eighteen linezolid-sensitive enterococci [50].

A noticeable polyclonal nature was observed in the *E. faecalis* isolates with 15 distinct STs, including one novel STs, highlighting the diverse nature of the strains in the province. The major STs found such as ST16, ST40, and ST21 were previously reported in Saudi Arabia, China, Tunisia, France, and Spain from human subjects, hospitalised patients, animals, and wastewater (Farman et al., 2019; [51–54]. Similarly, other studies have also reported ST126, ST23, and ST386 in different settings (human, animal, and environment) and hence do not suggest any kind of host specificity in these major STs reported in this study [55]. However, unlike other countries, the population structure *E. faecalis* from different settings in South Africa is minimally monitored, if at all, making it difficult to correlate our results with studies in South Africa. In a clonal analysis report of *E. faecalis* sourced from patients in a Chinese tertiary hospital, ST16 was predominantly present in urinary tract infections. In a Saudi-Arabian study, it accounted for the second most strain types and was also related to urinary tract infections. This calls for the need for *E. faecalis* to be included in surveillance schemes to enable the monitoring of the molecular epidemiology of isolates collected over larger tempo-spatial scales using high throughput technologies such as WGS [56]. Such surveillance would help microbiologists and public health practitioners to gain better insights into the evolution and dissemination of *E. faecalis*.

Characterizing the mobile genetic elements of the isolates indicated that the majority of *E. faecalis* in different hospitals are likely reservoirs for diverse mobile genetic elements and associated ARGs (especially for tetracycline and erythromycin). There was a higher plasmid prevalence rate (seven *rep* families) and the detection of two or more distinct replicons in one strain. Accordingly, this finding agrees with the fact that numerous types of plasmids are often present in enterococci in a clinical setting [57–59]. More so, other studies have shown that single isolates of *E. faecalis* may harbour multiple plasmids [57, 60].

There was no specific pattern between the acquisition of insertion sequence families or transposable elements with respect to the word and level of care; however, the presence of major FAMILIES in *E. faecalis* clones implies that these elements are spread by horizontal gene transfer (HGT) [35]. Moreover, the acquisition of these elements can lead to transposition in the genome aid in the transfer of resistance genes, enabling it to adapt to new environmental challenges and colonise new niches [61]. For instance, the IS3 family upstream of the *EmrB* gene has been reported for enhanced erythromycin resistance [61]. The ability of these clones to acquire novel genetic features may contribute to their increased persistence and highlights their potential public health threat.

Comparative phylogenomics using WGS SNPs analysis revealed a higher genetic diversity between the strains with respect to each specific hospital. This implied that the major clones were mostly hospital-specific, which was in concordance with the *in-silico* MLST typing scheme (Figure 1). Interestingly, a study by Kawalec et al. [62] also found a higher diversity in the clonal structure of *E. faecalis* strains among hospitals in Poland. Visualizing the phylogenomic tree with metadata revealed the major clones in various hospitals. This further depicted the intraclonal spread of *E. faecalis* strains between different sources within the same hospital, reiterating the need for phylo/meta-analysis to increase confidence in molecular epidemiological studies. For instance, at the paediatric ward of hospital A, the ST40 clone was isolated from a phone, nurse table, patient files, mop, and occupied bed, which may be due to hand contamination by patients and/or healthcare workers (nurses, janitor staff, etc.) (Figure 1). A similar scenario occurred in hospital B, where ST386 was found in the paediatric ward (on the phone, BP apparatus and unoccupied bed) while ST126 was isolated in the ICU (on the nurses' table and occupied bed). Reports on enterococci transient carriage on the hands of healthcare workers and patients as well as their presence on medical equipment or environmental surfaces have been documented in several studies [63–66]. Other studies have reported the movement of colonised patients among different settings in the hospital as responsible for these patterns of transmission [64, 67]. Moreover, hospitals B and C observed intraward spreads (both ICU and paediatric ward) of ST16 and 21, respectively, from different sites in each hospital. The transmission of enterococcal strains has been documented within medical units, giving credence to the study findings [68, 69].

Frequent contact with healthcare providers and movement of colonised patients among different healthcare settings are probable means for these patterns of transmission in hospitals A, B, and C. However, there were limited isolates from the district hospital (Hospital D) due to the number of isolates obtained for any detailed comparative analysis. Even though the findings of our study may not be generalised to the overall situation in the country, this study improves the understanding of the prevalence, genetic content, and relatedness of *E. faecalis* contamination in hospital environments. It is thus recommended that scheduled periodic identification of transmitting sources in hospitals' inanimate environment, strict enforcement and adhesion of IPC practices amongst health workers, and isolation of colonised patients should be imposed to reduce the incidence and transmission of *E. faecalis* in hospital environments. More so, the study was limited by the number of isolates selected for sequencing, and hence, there is a need for large-scale genomic epidemiology to elucidate the population structure in various hospital environments in South Africa.

## 5. Conclusion

This genomic analysis provided a snapshot of the hospital inanimate environment as a reservoir of resistant *E. faecalis*, its associated mobilome (plasmids, prophages, insertion sequences, and transposons), and revealed a complex intraclonal spread of *E. faecalis* major clones between the sites within each specific hospital setting. This study enhances our understanding of how *E. faecalis* spreads in hospital environments and investigates the role of the hospital environment in acquiring resistant genes, which poses a threat to the effective treatment of common infectious diseases. This will aid in the design of optimal infection prevention and control strategies in clinical settings. The findings may not represent the entire population of *E. faecalis* strains within the hospital settings; however, the observed trends in the major clones provide valuable preliminary information about their distribution and transmission patterns. These results can serve as a basis for further investigations and hypothesis generation in future studies.

## Figures and Tables

**Table 1:** Summary of the hospital levels, the source of sample collected, sample type, and genotypic characteristics of the *E. Faecalis* isolates.

Isolate ID	Sample details			Typing	Antibiotic resistance genes (ARGs)
	Hospital	Source	Ward	MLST (15)	
1MPA1	Central	PHONE	PEAD	ST40	*tetM, mphD, Isa(A)*
1MPA3	Central	PHONE	PEAD	ST40	*tetM, mphD, Isa(A)*
1MPD4	Central	PATIENT FILE	PEAD	ST16	*ermB, tetM, mphD, Isa(A), catA, dfrG, dfrK, Str*
1MPF1	Central	MOP	PEAD	ST498	*mphD, Isa(A)*
1MPF3	Central	MOP	PEAD	ST498-LIKE	*tetM, mphD, Isa(A)*
1MPJ101	Central	OCCUPIED BED	PEAD	ST40	*tetM, mphD, Isa(A)*
1MPK2	Central	NURSES TABLE	PEAD	ST40	*tetM, mphD, Isa(A)*
1MPK3	Central	NURSES TABLE	PEAD	ST40	*tetM, mphD, Isa(A)*
1MPK4	Central	NURSES TABLE	PEAD	ST40	*tetM, mphD, Isa(A)*
2MPJ104	Central	OCCUPIED BED	PEAD	ST23-LIKE	*tetM, mphD, Isa(A)*
3MPH1	Central	SINK	PEAD	ST610	*ermB, tetM, mphD, Isa(A), tetL*
3MPJ101	Central	OCCUPIED BED	PEAD	ST258	*mphD, Isa(A)*
2UIJ104	Tertiary	OCCUPIED BED	ICU	ST126	*tetM, mphD, Isa(A)*
2UIK2	Tertiary	NURSES TABLE	ICU	ST126	*tetM, mphD, Isa(A)*
2UIK3	Tertiary	NURSES TABLE	ICU	ST21	*ermB, tetM, mphD, Isa(A)*
2UPA3	Tertiary	PHONE	PEAD	ST386-LIKE	*mphD, Isa(A)*
2UPC4	Tertiary	BP APPARATUS	PEAD	ST386-LIKE	*mphD, Isa(A)*
2UPF4	Tertiary	MOP	PEAD	ST314	*mphD, Isa(A)*
2UPJ202	Tertiary	UNOCCUPIED BED	PEAD	ST386-LIKE	*mphD, Isa(A)*
3UIA2	Tertiary	PHONE	ICU	ST16	*tetM, mphD, Isa(A), catA, dfrG, Str*
3UIC1	Tertiary	BP APPARATUS	ICU	ST16	*ermB, tetM, mphD, Isa(A), catA, dfrG, sat4A, aph3-lll, ant6-la, aac6-Aph2*
3UIE2	Tertiary	VENTILATOR	ICU	ST268	*tetM, mphD, Isa(A)*
3UIJ202	Tertiary	UNOCCUPIED BED	ICU	ST282	*ermB,…, mphD, Isa(A), catA, dfrG, sat4A, aph3-lll, ant6-la, tetL*
3UPF3	Tertiary	MOP	PEAD	ST16	*ermB, tetM, mphD, Isa(A), dfrG*
3UPF4	Tertiary	MOP	PEAD	ST16	*ermB, tetM, mphD, Isa(A), dfrG*
3UPH1	Tertiary	SINK	PEAD	ST23	*tetM, mphD, Isa(A)*
3UIC2	Tertiary	BP APPARATUS	ICU	ST16	*ermB, tetM, mphD, Isa(A), catA, dfrG, sat4A,aAph3-lll, ant6-la, aac6-Aph2*
1CIB1	Regional	DRIP STAND	ICU	ST21	*ermB, tetM, mphD, Isa(A)*
1CID1	Regional	PATIENT FILE	ICU	ST21	*ermB, tetM, mphD, Isa(A)*
1CIH3	Regional	SINK	ICU	ST21	*ermB, tetM, mphD, Isa(A)*
1CPK2	Regional	NURSES TABLE	PEAD	ST21	*ermB, tetM, mphD, Isa(A)*
1CPK3	Regional	NURSES TABLE	PEAD	ST126	*tetM, mphD, Isa(A)*
2CPF3	Regional	MOP	PEAD	ST41	*tetM, mphD, Isa(A)*
2CPH2	Regional	NURSES TABLE	PEAD	ST16-LIKE	*ermB, tetM, mphD, Isa(A)*
3CPH1	Regional	SINK	PEAD	ST23	*tetM, mphD, Isa(A)*
2SIL2	District	DOOR HANDLE	ICU	ST922	*ermB, tetM, mphD, Isa(A),catA, dfrG, dfrK, sat4A, aph3-lll, ant6-la, tetL, fexA, optrA*
2SPJ101	District	OCCUPIED BED	ICU	ST6	*ermB, tetM, mphD, Isa(A), catA*
2SPL2	District	DOOR HANDLE	PEAD	ST314	*ermB, tetM, mphD, Isa(A)*

PEAD: paediatric ward; ICU: intensive care unit.

**Table 2:** Genomic analysis of mobile genetic elements (MGEs).

Strain ID	Hospital	MLST (*n* = 15)	Mobile genetic support	
			Plasmids (plasmid replicons) (*n* = 11)	Intact prophage (*n* = 18)
1MPA1	Central	ST40	pTEF3 (repUS13), pAD1 (rep9), pEF47 (rep6)	Entero_phiFL1A
1MPA3	Central	ST40	pTEF3 (repUS13), pAD1 (rep9), pEFC1 (rep6)	Entero_phiFL1A
1MPD4	Central	ST16	—	Entero_phiFL1A, Entero_EFC_1, Lactob_PLE2
1MPF1	Central	ST498	pTEF3 (repUS13)	Entero_phiFL3A
1MPF3	Central	ST498	pTEF3 (repUS13), pAD1 (rep9), pEFC1 (rep6)	Entero_phiFL3A, Entero_phiFL1A
1MPJ101	Central	ST40	pTEF3 (repUS13), pAD1 (rep9), pEFC1 (rep6)	Entero_phiFL1A
1MPK2	Central	ST40	pTEF3 (repUS13), pAD1 (rep9), pEFC1 (rep6)	Entero_phiFL1A
1MPK3	Central	ST40	pTEF3 (repUS13), pAD1 (rep9), pEFC1 (rep6)	Entero_phiFL1A
1MPK4	Central	ST40	pTEF3 (repUS13), pAD1 (rep9), pEFC1 (rep6)	Entero_phiFL1A
2MPJ104	Central	ST23	pAD1 (rep9), pEF47 (rep6)	Entero_phiFL3A, Entero_phiFL1A
3MPH1	Central	ST610	pTEF2 (rep9)	—
3MPJ101	Central	ST258	pTEF2 (rep9)	Strept_9871
2UIJ104	Tertiary	ST126	pTEF2 (rep9), pAD1 (rep9)	Entero_phiFL1A, Lactoc_98201
2UIK2	Tertiary	ST126	pTEF2 (rep9), pAD1 (rep9)	Entero_phiFL1A, Lactoc_98201, Cronob_vB_CsaM
2UIK3	Tertiary	ST21	pTEF2 (rep9)	Entero_vB_IME197, Lactoc_63301
2UPA3	Tertiary	ST386	pTEF3 (repUS13), pEFC1 (rep6)	Entero_phiEf11
2UPC4	Tertiary	ST386	pTEF3 (repUS13), pEF47 (rep6)	Entero_phiEf11
2UPF4	Tertiary	ST314	—	—
2UPJ202	Tertiary	ST386	pTEF3 (repUS13), pEFC1 (rep6)	Entero_phiEf11
3UIA2	Tertiary	ST16	—	Entero_EF62phi, Strept_phiARI0460_1
3UIC1	Tertiary	ST16	pTEF2 (rep9), pCF10 (rep9)	Entero_phiEf11
3UIE2	Tertiary	ST268	—	Entero_phiFL1A
3UIJ202	Tertiary	ST282	pTEF2 (rep9), pAD1 (rep9)	—
3UPF3	Tertiary	ST16	—	Entero_EFC_1, Strept_phiARI0131_1, Strept_phiARI0460_1
3UPF4	Tertiary	ST16	—	Entero_phiFL1A, Entero_EFC_1, Strept_phiARI0460_1, Lactoc_PLgT_1
3UPH1	Tertiary	ST23	pEFC1 (rep6)	Entero_phiFL3A
3UIC2	Tertiary	ST16	pTEF2 (rep9), pCF10 (rep9)	Entero_phiFL1A, Entero_phiEf11
1CIB1	Regional	ST21	pTEF2 (rep9)	Entero_vB_IME197, Lactoc_63301
1CID1	Regional	ST21	pTEF2 (rep9)	Entero_vB_IME197, Lactob_PLE2
1CIH3	Regional	ST21	pTEF2 (rep9)	Entero_vB_IME197, Lactoc_63301, Stx2_c_1717
1CPK2	Regional	ST21	pTEF2 (rep9)	Entero_vB_IME197, Lactob_PLE2
1CPK3	Regional	ST126	pTEF2 (rep9), pAD1 (rep9)	Lactoc_98201
2CPF3	Regional	ST41	pTEF3 (repUS13)	Entero_vB_IME197, Lactob_PLE2
2CPH2	Regional	ST16	—	Entero_phiFL1A
3CPH1	Regional	ST23	—	Entero_phiFL3A, Entero_phiFL1A
2SIL2	District	ST922	pk214 (rep7), pAD1 (rep9), pEFR (rep11)	Entero_phiFL3A, Paenib_Xenia
2SPJ101	District	ST6	pTEF3 (repUS13), pPD1 (rep9), pRE25 (rep2), pUB110 (repUS14), pKH7 (rep7)	Entero_SANTOR1
2SPL2	District	ST314	pTEF2 (rep9)	Strept_9872

NB. All the isolates lacked integrons and associated gene cassettes.

## Data Availability

The generated data used to support the findings of this study are included in the article.

## Supplementary Materials

Supplementary Materials Table S1: List of genus- and species-specific primers and control strains used in this study. Table S2: Antibiotic susceptibility profiles of *E. faecalis* (*n* = 38). Table S3: Distribution of 6 major insertion sequence (IS)/transposase families and their associated predicted sources among *E. faecalis* isolates via the ISFinder database.
