# Supplementary material for: A Genomic Snapshot of Antibiotic-ResistantEnterococcus faecalis within Public Hospital Environments in South Africa
Source: Glob Health Epidemiol Genom. 2023 Jun 12;2023:6639983. doi: 10.1155/2023/6639983 (PMC10279497; doi:10.1155/2023/6639983)
Supplement: Supplementary Materials — Table S1: List of genus- and species-specific primers and control strains used in this study. Table S2: Antibiotic susceptibility profiles of E. faecalis (n = 38). Table S3: Distribution of 6 major insertion sequence (IS)/transposase families and their associated predicted sources among E. faecalis isolates via the ISFinder database. [file 6639983.f1.docx]

**SUPPLEMENTARY MATERIAL**

**A Genomic Snapshot of Antibiotic Resistant Enterococcus faecalis within Public Hospital Environments in South Africa**

Christiana O. Shobo^1, 2, 3^, Daniel G. Amoako^1, 3, 4*^, Mushal Allam^4^, Arshad Ismail^4^, Sabiha Y. Essack^1^, Linda A. Bester^3*^

^1^Antimicrobial Research Unit, College of Health Sciences, University of KwaZulu-Natal, Durban, South Africa;

^2^School of Laboratory Medicine and Medical Science, Department of Medical Microbiology, University of KwaZulu-Natal, Durban, South Africa;

^3^Biomedical Resource Unit, School of Laboratory Medicine and Medical Sciences, College of Health Sciences, University of KwaZulu-Natal; Durban, South Africa;

^4^Sequencing Core Facility, National Institute for Communicable Diseases, National Health Laboratory Service, Johannesburg, South Africa;

*Corresponding Authors: Linda A. Bester (Ph.D.), [besterl@ukzn.ac.za](mailto:besterl@ukzn.ac.zam) (https://orcid.org/0000-0001-5726-681X) and Daniel G. Amoako (Ph.D.), **Email:** amoakodg@gmail.com **ORCID**: <http://orcid.org/0000-0003-3551-3458> Tel.: +27(0) +27 (0) 843308957.

**Table S1:** List of genus-and species-specific primers and control strains used in this study

| Control Strain | Primer | Primer sequence 5’-3’ | Product size (bp) | Reference |
| --- | --- | --- | --- | --- |
| *E. faecalis* ATCC 51299 | ENT1  ENT2 | TACTGACAAACCATTCATGATG  AACTTCGTCACCAACGCGAAC | 112 | (Molechan et al., 2019) |
| *E. faecalis* ATCC 51299 | FA1  FA2 | ACTTATGTGACTAACTTAACC  TAATGGTGAATCTTGGTTTGG | 360 | (Molechan et al., 2019) |

**Table S2:** Antibiotic susceptibility profiles of *E. faecalis*

^a^. Antibiotic susceptibility tests were interpreted according to CLSI resistant breakpoints (v 7.1) for *E. faecalis*.

| Isolate | \|  \|  \| Antibiotic susceptibility testing ^a^ \| \| --- \| --- \| --- \| | | | | | | | | | | | | | |
| --- | --- | --- | --- | --- | --- | --- | --- | --- | --- | --- | --- | --- | --- | --- | --- | --- | --- |
| Strain ID | VAN | TEC | CIP | LEV | GEN | STP | TET | ERY | CHLO | RIF | F300 | LZD | PEN | AMP |
| 1MPA1 | I | I | I | S | S | S | R | I | S | I | S | S | S | S |
| 1MPA3 | I | S | I | S | S | S | R | I | S | I | S | S | S | S |
| 1MPD4 | I | S | I | S | S | R | R | R | R | R | S | R | S | S |
| 1MPF1 | I | S | R | S | S | S | S | I | S | R | S | I | S | S |
| 1MPF3 | I | S | R | S | S | S | R | I | S | I | S | R | S | S |
| 1MPJ101 | I | S | S | S | S | S | R | I | S | I | S | R | S | S |
| 1MPK2 | I | I | I | S | S | S | R | I | S | I | S | S | S | S |
| 1MPK3 | I | S | S | S | S | S | I | I | S | I | S | S | S | S |
| 1MPK4 | I | S | I | S | S | S | R | I | S | I | S | S | S | S |
| 2MPJ104 | I | S | I | S | S | S | R | I | S | I | S | I | S | S |
| 3MPH1 | I | S | R | S | S | S | R | R | S | I | S | S | S | S |
| 3MPJ101 | I | S | I | S | S | S | S | I | I | R | S | R | S | S |
| 2UIJ104 | I | S | R | S | S | S | R | I | S | R | S | R | S | S |
| 2UIK2 | I | S | I | S | S | S | R | I | S | R | S | R | S | R |
| 2UIK3 | I | S | I | I | S | S | R | R | S | I | S | S | S | S |
| 2UPA3 | I | S | R | I | S | S | I | R | R | S | S | I | S | S |
| 2UPC4 | I | S | I | I | S | S | I | I | S | S | S | S | S | S |
| 2UPF4 | I | S | I | S | S | S | I | I | I | R | I | R | S | S |
| 2UPJ202 | I | S | I | S | S | S | S | I | S | I | S | S | S | S |
| 3UIA2 | I | S | I | S | S | S | R | I | R | R | S | S | S | S |
| 3UIC1 | I | S | I | S | R | R | R | R | R | R | S | S | S | S |
| 3UIE2 | I | S | I | S | S | S | R | I | I | R | S | R | S | S |
| 3UIJ202 | I | S | I | S | R | R | S | R | R | R | S | R | S | S |
| 3UPF3 | I | S | S | S | S | S | R | R | S | R | S | R | S | S |
| 3UPF4 | I | S | R | I | S | S | R | R | S | R | S | R | R | S |
| 3UPH1 | I | S | I | I | S | S | R | I | I | R | S | R | S | S |
| 3UIC2 | I | S | I | I | R | R | R | R | R | R | S | I | S | S |
| 1CIB1 | I | S | I | R | S | S | R | R | I | R | S | S | S | S |
| 1CID1 | I | S | S | S | S | S | R | R | S | R | S | R | S | S |
| 1CIH3 | I | S | R | S | S | S | R | R | S | R | S | S | S | S |
| 1CPK2 | I | S | I | S | S | S | R | R | I | I | S | S | S | S |
| 1CPK3 | I | S | S | S | S | S | R | R | I | I | S | S | S | S |
| 2CPF3 | I | S | I | S | S | S | R | I | I | I | S | R | S | S |
| 2CPH2 | I | S | I | S | S | S | R | R | I | R | S | R | S | S |
| 3CPH1 | I | S | I | S | S | S | R | I | I | R | S | S | S | S |
| 2SPJ101 | I | S | I | S | S | S | R | R | R | I | S | R | S | S |
| 2SPL2 | I | S | I | S | S | S | R | R | R | I | S | R | S | S |
| 2SIL2 | I | S | S | S | R | R | R | R | R | R | S | S | S | S |

**Glycopeptides**: VAN = Vancomycin, TEC = Teicoplanin;

**Quinolones**: CIP =ciprofloxacin, LEV = levofloxacin;

**Aminoglycosides**: STP = streptomycin, GEN = gentamycin;

**Tetracyclines**: TET = tetracycline;

**Macrolides**: ERY = erythromycin;

**Amphenicols**: CHLO = chloramphenicol;

**Rifamycin**: RIF = rifampicin;

**Nitrofurans**: F300 = nitrofurantoin;

**Oxazolidinones**: LZD = linezolid;

**Penicillin**: PEN = penicillin G; AMP = ampicillin.

**R, S and I =** Resistant, Susceptible and Intermediate respectively.

**Table S3:** Distribution of 6 major insertion sequence (IS)/transposase families and their associated predicted sources among *E. faecalis* isolates via the ISFinder database.

| IS Family | Number of occurrences | Predicted Sources |
| --- | --- | --- |
| IS3 | *21* | *Enterococcus faecium*  *Streptococcus agalactiae* |
| IS5 | *16* | *Cyanotheca sp.* |
| IS1595 | *15* | *Bacillus subtilis* |
| ISL3 | *9* | *Streptococcus mutans*  *Streptococcus thermophilus* |
| IS607 | *9* | *Campylobacter sp.*  *Virus NY2A* |
| Tn3 | *7* | *Bacillus thuringiensis* |
